# NEIGHBORHOOD AND DEPRESSION AMONG US CHINESE OLDER IMMIGRANTS: THE MEDIATION EFFECTS OF COPING

**DOI:** 10.1093/geroni/igad104.1412

**Published:** 2023-12-21

**Authors:** Yi Wang, Man Guo, Jinyu Liu, Yifan Lou, Kara Carter

**Affiliations:** University of Iowa, Iowa City, Iowa, United States; University of Iowa, Iowa City, Iowa, United States; Baylor University, Waco, Texas, United States; Yale University, New Haven, Connecticut, United States; University of Iowa, Iowa City, Iowa, United States

## Abstract

Studies have shown that neighborhood environment shapes older Americans’ aging experience and health. However, it remains largely unknown whether and how neighborhood environment influences the well-being of older Asian Immigrants. Guided by the neighborhood stress process model, this study aims to investigate (a) the associations between neighborhood environmental stressors and depression among Chinese older immigrants and (b) the potential mediation effects of intrapersonal (sense of mastery and sense of hopefulness) and interpersonal coping (social engagement) resources in such associations. This study analyzed data collected from 2,801 Chinese older immigrants in the greater Chicago area. Structural equation modeling with bootstrap resampling was used to fit path models on neighborhood environmental stressor, intra- and interpersonal coping resources, and depression. Findings showed that neighborhood social disintegration and physical disorder were associated with more depressive symptoms directly and indirectly via lower intra- and interpersonal coping resources. Specifically, older immigrants living in neighborhoods with greater social disintegration reported lower sense of mastery and social engagement, which in turn were associated with more depressive symptoms (partial mediation). Older immigrants living in neighborhoods with greater physical disorder reported lower sense of hopefulness and mastery, which subsequently were associated with more depressive symptoms (full mediation). The findings showed that neighborhood environmental stressors are risk factors for mental health of Chinese older immigrants, and coping resources may serve as pathways of the associations. The findings have implications for practice as they are helpful to inform the development of tailored psychosocial interventions to enhance coping resources in older immigrants.

